# Supplementary figures and images for: A priority experience replay actor-critic algorithm using self-attention mechanism for strategy optimization of discrete problems
Source: PeerJ Comput Sci. 2024 Jun 28;10:e2161. doi: 10.7717/peerj-cs.2161 (PMC11232580; doi:10.7717/peerj-cs.2161)

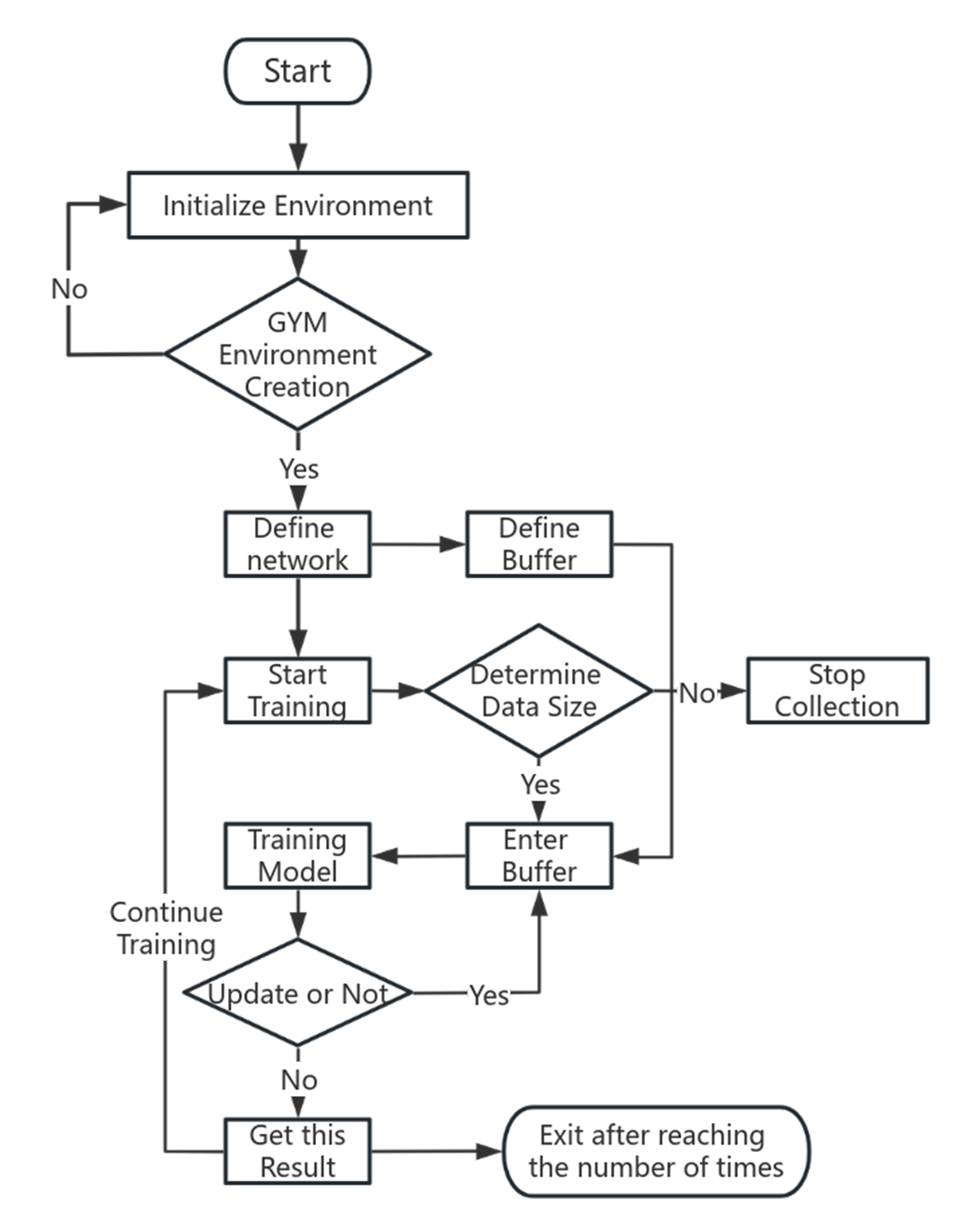

Supplement: Supplemental Information 1 [file peerj-cs-10-2161-s001.png]

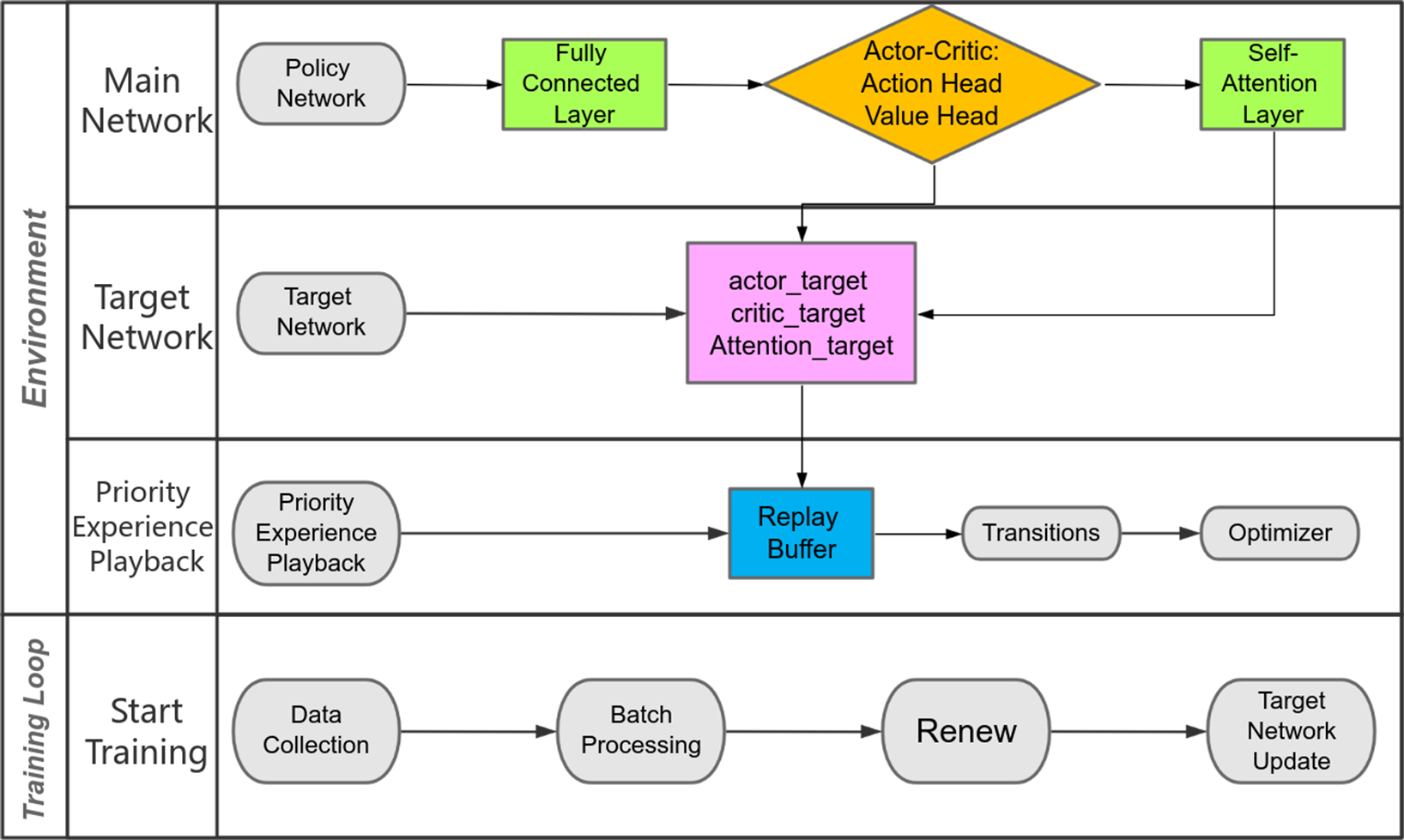

Supplement: Supplemental Information 2 [file peerj-cs-10-2161-s002.png]
