# Supplementary material for: A priority experience replay actor-critic algorithm using self-attention mechanism for strategy optimization of discrete problems
Source: PeerJ Comput Sci. 2024 Jun 28;10:e2161. doi: 10.7717/peerj-cs.2161 (PMC11232580; doi:10.7717/peerj-cs.2161)
Supplement: Supplemental Information 3 [file peerj-cs-10-2161-s003.docx]

**Appendix C: Pseudocode of the A2CPER algorithm**

| **Pseudocode of A2CPER Algorithm: (eg:CartPole-v1**) |
| --- |
| 0: repeat |
| 1: procedure PRIORITIZED_ACTOR_CRITIC_SELF_ATTENTION(CartPole-v1) |
| 2: Initialize env ← gym.make('CartPole-v1').unwrapped |
| 3: Set seed for reproducibility: env. seed(1), torch.manual_seed(1) |
| 4: Define state_space, action_space, MEMORY_CAPACITY, batch_size,  $\gamma$, episodes |
| 5: Initialize Policy Network with Self-Attention (state_space, action_space) |
| 6: optimizer ← Adam(policy.parameters, lr = 0.01) |
| 7: prioritized_replay_buffer ←  PrioritizedReplayBuffer(MEMORY_CAPACITY, $\alpha$) |
| 8: for i_episode ∈ {1, 2, ..., episodes} do |
| 9: state ← env. reset() |
| 10: for t ← 1 to T do |
| 11: $\pi(a\vert s), V(s)$← policy(state) |
| 12: action ← sample($\pi(a\vert s)$) |
| 13: next_state, reward, done ← env.step(action) |
| 14: error ← $\vert r + \gamma* V(s') - V(s)\vert$ |
| 15: prioritized_replay_buffer.store(  (state, action, reward, next_state, done), error) |
| 16: if len(prioritized_replay_buffer) ≥ batch_size then |
| 17: B, indices, weights ← prioritized_replay_buffer.sample(batch_size) |
| 18: for each idx ∈ indices do |
| 19: $s, a, r, s', d$ ← B[idx] |
| 20: Q_targets ←  $r + \gamma* (1 - d) * min(Q\_policy(s', a') - \beta* N(s'))$ |
| 21: $\Delta\theta$ ← $weights[idx] * (\pi(a\vert s), V(s) - Q\_targets)^2$ |
| 22: policy_loss ← policy_loss + Δθ |
| 23: end for |
| 24: Update policy using gradient descent: ∇_θ J(θ) |
| 25: Update priorities in prioritized_replay_buffer for indices |
| 26: end if |
| 27: if i_episode mod plot_frequency = 0 then |
| 28: PlotProgress(episode_rewards) |
| 29: end for |
| 30: Evaluate policy by testing it on the environment. |
| 31: end procedure |
