# Supplementary material for: A priority experience replay actor-critic algorithm using self-attention mechanism for strategy optimization of discrete problems
Source: PeerJ Comput Sci. 2024 Jun 28;10:e2161. doi: 10.7717/peerj-cs.2161 (PMC11232580; doi:10.7717/peerj-cs.2161)
Supplement: Supplemental Information 4 [file peerj-cs-10-2161-s004.docx]

**Appendix D: Hyperparameters used in experiments**

| **Hyperparameter** | **Values** | **Comment** |
| --- | --- | --- |
| batch_size | 128 | Number of transitions sampled from replay buffer per training batch. |
| MEMORY_CAPACITY | 200,000 | Maximum capacity of the experience replay buffer. |
| learning_rate | 0.01 | Step size used by the optimizer for updating the weights. |
| gamma | 0.99 | Discount factor for computing the present value of future rewards. |
| episodes | 2,000 | Total number of episodes for the training process. |
| tau | 0.001 | Soft update rate for the target network parameters. |
| eps | 1.19209e-07 | Epsilon is used to prevent division by zero or very small values in numerical operations. |
| epsilon_start | 1.0 | Initial value of the exploration rate for the epsilon-greedy strategy. |
| epsilon_final | 0.01 | Final exploration rate at the end of training. |
| epsilon_decay | 0.995 | The decay rate of the exploration rate over time. |
| alpha | 0.6 - 0.7 | Exponent of prioritization in Prioritized Experience Replay, determining the importance of TD error. |
| beta | 0.4 - 0.5 | Exponent of importance sampling in Prioritized Experience Replay, starting from this value and annealing to 1 |
| update_target | 1,000 | Number of steps between updates of the target network weights. |
| max_grad_norm | 0.5 - 1 | Maximum gradient norm for gradient clipping. |
| reward_scaling | 1.0 | Scaling factor for rewards to control the magnitude and impact on the gradient scale. |
| learning_starts | 1,000 | A number of timesteps before learning starts to initially populate the replay buffer. |
